# The Influence of Regional Anesthesia on the Systemic Stress Response

**DOI:** 10.3390/reports7040089

**Published:** 2024-11-02

**Authors:** Tomasz Reysner, Katarzyna Wieczorowska-Tobis, Grzegorz Kowalski, Monika Grochowicka, Monika Pyszczorska, Aleksander Mularski, Malgorzata Reysner

**Affiliations:** 1 Department of Palliative Medicine Poznan, University of Medical Sciences, 61-245 Poznań, Poland; 2 Department of Forensic Medicine, Institute of Medical Sciences Collegium Medicum, University of Zielona Góra, 65-417 Zielona Góra, Poland

**Keywords:** NLR, PLR, CRP, nerve block, cytokines, TNF-α, systemic inflammatory response syndrome, spinal aesthesia, epidural aneshtesia, stress responce

## Abstract

**Background:** The systemic stress response to surgery is a complex physiological process characterized by neuroendocrine, sympathetic, and inflammatory activation. While necessary for survival, this response can lead to adverse outcomes such as hyperglycemia, immune suppression, cardiovascular complications, and delayed recovery. Regional anesthesia (RA) has been shown to modulate this stress response more effectively than general anesthesia (GA) by blocking nociceptive signaling and attenuating the release of stress mediators. **Objectives:** This review aims to elucidate how RA influences the systemic stress response, highlighting its clinical benefits in reducing postoperative pain, improving hemodynamic stability, minimizing inflammatory responses, and preserving immune function. Additionally, this review examines evidence from clinical trials supporting using RA to improve surgical outcomes, particularly in high-risk populations. **Methods:** A comprehensive narrative review of the literature was conducted to explore the physiological impact of RA on the systemic stress response and its associated clinical outcomes. Studies comparing RA to GA across various surgical procedures were evaluated, focusing on neuroendocrine modulation, sympathetic inhibition, inflammatory attenuation, and the implications for pain management, cardiovascular and pulmonary function, and immune preservation. **Results:** RA significantly attenuates the neuroendocrine response by reducing the release of cortisol and catecholamines, thereby improving hemodynamic stability and reducing myocardial oxygen consumption. RA also inhibits the sympathetic nervous system, leading to improved cardiovascular outcomes. Furthermore, RA mitigates the inflammatory response by reducing pro-inflammatory cytokine levels, reducing the risk of systemic inflammatory response syndrome (SIRS), sepsis, and pulmonary complications. Clinical studies and meta-analyses consistently demonstrate that RA reduces postoperative pain, opioid consumption, and the incidence of cardiovascular and pulmonary complications, particularly in elderly and high-risk patients. **Conclusions:** RA offers a significant advantage in modulating the systemic stress response to surgery, improving postoperative outcomes by reducing pain, enhancing cardiovascular stability, and preserving immune function. Its benefits are particularly pronounced in high-risk populations such as the elderly or those with pre-existing comorbidities. Given the growing evidence supporting its efficacy, RA should be considered a critical component of multimodal perioperative care strategies aimed at minimizing the systemic stress response and improving recovery. Future research should optimize RA techniques and identify patient-specific factors to enhance therapeutic benefits.

## 1. Introduction

Surgical stress is a profound physiological disturbance triggered by tissue injury, anesthesia, and other perioperative factors [1]. This stress response encompasses a complex interplay of neuroendocrine, immunological, and metabolic pathways [2]. It is characterized by activating the hypothalamic–pituitary–adrenal (HPA) axis, the sympathetic nervous system (SNS), and the release of inflammatory cytokines [3]. While the systemic stress response is a survival mechanism, excessive activation may result in adverse outcomes, including hyperglycemia, immune dysfunction, increased cardiovascular demand, and impaired wound healing [4,5]. These sequelae can prolong hospital stays, increase morbidity, and lead to significant postoperative complications [6].

Regional anesthesia (RA) has gained recognition for modulating the systemic stress response through its ability to block afferent neural pathways [7]. In contrast to general anesthesia (GA), which does little to attenuate neurohormonal responses, RA reduces nociceptive transmission at the injury site, limiting the cascade of neuroendocrine and inflammatory responses [8]. This review delves into the physiological mechanisms by which RA influences the systemic stress response, reviews key clinical evidence supporting its use, and discusses the broader implications for perioperative care.

## 2. Study Methodology

This narrative review followed an inductive approach to explore the impact of RA on the systemic stress response in surgical settings, formulating insights based on the synthesis of the existing literature.

The primary research question was: how does regional anesthesia influence the physiological stress response, particularly compared to general anesthesia, regarding inflammation, neuroendocrine modulation, and clinical outcomes?

### Literature Search Strategy

We conducted a systematic literature search using the PubMed, MEDLINE, and Cochrane Library databases to identify peer-reviewed articles published from 2010 to 2024. We included clinical studies, systematic reviews, and meta-analyses focused on the effects of RA and GA on the surgical stress response. The following search terms were used: “regional anesthesia”, “surgical stress response”, “general anesthesia”, “inflammatory markers”, “neuroendocrine response”, “mortality”, “ICU length of stay,” and “major complications”. Articles were screened by title, abstract, and relevance, followed by a full-text review based on their methodology, relevance to the study question, and quality.

## 3. The Surgical Stress Response: A Physiological Overview

The surgical stress response involves three major systems [9]: the neuroendocrine, sympathetic, and immune systems.

### 3.1. Neuroendocrine Response

Surgical trauma initiates a cascade beginning with the release of corticotropin-releasing hormone (CRH) from the hypothalamus [2]. This, in turn, stimulates the release of adrenocorticotropic hormone (ACTH) from the anterior pituitary, which drives the adrenal glands to secrete cortisol, the primary stress hormone [10]. Cortisol exerts broad metabolic effects, including gluconeogenesis, proteolysis, and lipolysis, all aimed at mobilizing energy substrates to cope with the trauma [11]. However, prolonged elevation of cortisol levels can lead to hyperglycemia, insulin resistance, and increased susceptibility to infections [12].

### 3.2. Sympathetic Nervous System (SNS) Activation

Simultaneously, the SNS is activated, releasing catecholamines—primarily adrenaline and noradrenaline [13]. These catecholamines increase heart rate, contractility, and systemic vascular resistance, preparing the body for a fight-or-flight response [8]. While this cardiovascular adjustment is adaptive in the acute phase, prolonged catecholamine exposure can contribute to cardiac ischemia, arrhythmias, and increased oxygen consumption [14].

### 3.3. Inflammatory Response

Tissue injury activates the immune system by releasing pro-inflammatory cytokines, such as interleukin (IL)-1, IL-6, and tumor necrosis factor-alpha (TNF-α). These cytokines trigger the acute phase response, promoting the production of acute phase proteins like C-reactive protein (CRP) and fibrinogen [15]. In parallel, neutrophils are rapidly recruited to the site of injury as the first line of defense, where they play a crucial role in phagocytosis and releasing reactive oxygen species to combat infection [16]. Platelets also facilitate clot formation and release signaling molecules that amplify the inflammatory response [17]. Lymphocytes, particularly T-cells, become involved later, contributing to the adaptive immune response and promoting tissue repair [18].

While this inflammatory response is essential for controlling infection and promoting wound healing, excessive activation can result in dysregulated immune function [19]. The overproduction of cytokines, excessive neutrophil activation, and platelet aggregation can drive systemic inflammatory response syndrome (SIRS), leading to endothelial damage, capillary leak, and multiorgan dysfunction [20]. Persistent lymphocyte activation can also exacerbate systemic inflammation, further heightening the risk of sepsis and poor postoperative outcomes [21]. Thus, controlling this immune response is critical to avoiding these severe complications [22].

## 4. Mechanisms by Which Regional Anesthesia Attenuates the Systemic Stress Response

### 4.1. Neuroendocrine Modulation

RA interrupts the afferent neural signals responsible for triggering the neuroendocrine stress response [23]. By blocking nerve impulses from the site of surgical injury, RA effectively reduces the release of CRH and dampens the HPA axis [7], resulting in lower circulating ACTH and cortisol [10]. Neuraxial techniques such as spinal and epidural anesthesia are particularly effective at inhibiting the neuroendocrine response [24]. For instance, studies have shown that epidural and anesthesia can significantly reduce plasma cortisol levels in patients undergoing central abdominal surgery [25].

Beyond its effects on cortisol, RA also blunts the stress-induced secretion of catecholamines [26]. Reducing noradrenaline and adrenaline levels improves hemodynamic stability, reduces myocardial oxygen consumption, and lowers the incidence of arrhythmias [27]. This is especially advantageous in elderly patients or those with cardiovascular disease, where exaggerated neuroendocrine responses may precipitate cardiac complications [28].

### 4.2. Sympathetic Nervous System (SNS) Inhibition

A critical stress response component involves activating the SNS, which mediates vasoconstriction, tachycardia, and increased blood pressure [29]. The administration of RA, particularly neuraxial blocks, inhibits sympathetic outflow by blocking the transmission of nociceptive stimuli at the spinal level [30]. This results in a significant reduction in the release of catecholamines [31]. Thoracic epidural anesthesia, in particular, has been shown to modulate cardiovascular stress by blocking the sympathetic chain at the thoracic level, reducing heart rate and blood pressure, and improving myocardial oxygen supply-demand balance [32]. Peripheral nerve blocks, such as the femoral and sciatic nerve blocks commonly used in orthopedic surgeries, also provide sympathetic blockade within their respective dermatomes, reducing peripheral vascular resistance and decreasing the systemic catecholamine surge [33].

### 4.3. Inflammatory Modulation

Surgical trauma induces a local and systemic inflammatory response, marked by the release of pro-inflammatory cytokines such as IL-6, IL-8, and TNF-α. RA, by blocking nociceptive input at the site of surgery, can mitigate the inflammatory cascade [34]. Several clinical studies have demonstrated this reduction in cytokine release, showing that patients receiving RA exhibit significantly lower postoperative levels of IL-6, IL-8, TNF-α, and CRP than those undergoing GA alone [35,36,37]. A reduction in pro-inflammatory cytokines has multiple clinical benefits. It attenuates the acute phase response and reduces the risk of postoperative complications such as sepsis and SIRS [38]. Moreover, by dampening the inflammatory response, RA may accelerate wound healing and reduce postoperative pain, further contributing to improved recovery [39].

## 5. Clinical Implications of Regional Anesthesia on the Systemic Stress Response

### 5.1. Impact on Postoperative Pain and Opioid Consumption

Adequate postoperative analgesia is crucial for mitigating the systemic stress response [40]. Poorly controlled pain results in persistent activation of the HPA axis and SNS, prolonging the release of stress hormones and inflammatory mediators [41]. RA provides superior pain control to systemic analgesics, directly interrupting nociceptive transmission [42]. Consequently, patients who receive RA typically report lower postoperative pain scores and have a reduced need for opioid analgesics [43]. By reducing opioid consumption, RA also minimizes the risk of opioid-related side effects such as respiratory depression, nausea, constipation, and opioid-induced hyperalgesia [44]. This opioid-sparing effect is particularly beneficial in elderly patients and those with pre-existing respiratory or cardiovascular conditions [45].

### 5.2. Cardiovascular Stability and Reduced Myocardial Stress

One of the most well-established benefits of RA is its ability to reduce perioperative cardiovascular complications [46]. By attenuating the release of catecholamines, RA reduces myocardial oxygen demand, limits tachycardia, and stabilizes blood pressure [47]. This is particularly important in high-risk patients undergoing major surgeries, such as those with pre-existing coronary artery disease or undergoing vascular surgery [27]. In these patients, the reduction in catecholamine release associated with RA may lower the risk of myocardial infarction, arrhythmias, and heart failure [48]. In addition, neuraxial blocks such as thoracic epidurals have been associated with improved myocardial perfusion, as they reduce afterload and increase coronary blood flow [27]. Several studies have demonstrated that patients receiving epidural anesthesia exhibit a reduced incidence of perioperative myocardial ischemia and infarction [49].

### 5.3. Pulmonary Function and Respiratory Complications

The stress response can impair pulmonary function through multiple mechanisms, including increased respiratory rate, decreased lung compliance, and the development of atelectasis [50]. By reducing pain and cytokine release, RA helps preserve respiratory function, reducing the risk of postoperative pulmonary complications such as pneumonia, atelectasis, and acute respiratory distress syndrome (ARDS) [51]. Thoracic epidural anesthesia, in particular, has been shown to improve postoperative lung function by providing superior pain relief and reducing the need for systemic opioids, which can depress respiratory drive [52]. In patients undergoing major abdominal or thoracic surgeries, thoracic epidural blocks and peripheral nerve blocks have been associated with a lower incidence of pulmonary complications and a shorter time to extubation [53].

### 5.4. Immunological Preservation

The immunosuppressive effects of surgery and anesthesia are well-documented and are primarily mediated by cortisol and catecholamines [54]. Prolonged immunosuppression can increase the risk of infections, impair wound healing, and facilitate tumor metastasis in cancer patients [55]. By reducing the neuroendocrine stress response, RA helps preserve immune function, as evidenced by higher postoperative levels of natural killer (NK) cell activity and lower infection rates in patients receiving RA compared to GA [56].

### 5.5. Impact on Special Populations: The Elderly and High-Risk Patients

Due to physiological frailty and a high prevalence of comorbidities, the elderly are particularly vulnerable to the harmful effects of the systemic stress response [57]. In this population, RA has been shown to reduce the incidence of postoperative delirium, improve pain control, and lower the risk of cardiovascular and pulmonary complications [8]. In orthopedic surgeries such as hip or knee arthroplasty, RA has been associated with improved functional recovery, shorter hospital stays, and reduced 30-day mortality [58].

## 6. Evidence from Clinical Trials

Regional anesthesia (RA) has emerged as a pivotal modality in the perioperative management of surgical patients, mainly due to its ability to modulate the systemic stress response elicited by surgery. Numerous clinical studies have demonstrated that RA not only provides adequate analgesia but also exerts profound effects on physiological markers associated with surgical stress, such as inflammatory and oxidative stress markers [59,60,61]. These effects are particularly evident in reducing inflammatory markers, such as the neutrophil-to-lymphocyte ratio (NLR) and the platelet-to-lymphocyte ratio (PLR), as well as stress hormones, including cortisol and adrenaline [58].

The NLR and PLR have been recognized as reliable indicators of systemic inflammation and stress. Elevations in these ratios are expected in response to surgical trauma, reflecting both an inflammatory and immune response. In randomized controlled trials (RCTs), the application of RA, mainly through peripheral nerve blocks and fascial plane blocks, has consistently attenuated these markers. Domagalska et al. [22] conducted a randomized trial in a cohort of 100 pediatric patients undergoing idiopathic scoliosis correction surgery. They found that using an Erector Spinae Plane Block (ESPB) in conjunction with general anesthesia significantly reduced both NLR and PLR levels postoperatively. This reduction suggests a decrease in the systemic inflammatory burden, potentially leading to improved postoperative recovery and decreased risk of complications associated with heightened inflammatory responses, such as infection and delayed healing.

Similarly, Chen et al. [62] demonstrated that using a paravertebral block guided by ultrasound significantly lowered NLR values in patients undergoing thoracic surgery. This reinforces the concept that RA, mainly when applied to regions of high surgical stress, can mitigate the inflammatory response. These findings underscore the ability of RA to not only provide regional analgesia but also to blunt the systemic consequences of surgical trauma, a key consideration in enhancing postoperative outcomes. Interestingly, despite these benefits, it has been noted that the addition of adjuvants to local anesthetics does not appear to influence NLR or PLR levels when used in conjunction with peripheral nerve blocks [63], indicating that the primary impact of RA on these markers may be driven by the mechanical and physiological blockade of nerve pathways, rather than pharmacological modulation.

RA can significantly impact oxidative stress levels, which is a crucial aspect of the surgical stress response [64]. Oxidative stress, marked by the overproduction of reactive oxygen species (ROS) like superoxide, contributes to cellular damage, inflammation, and delayed wound healing [65]. During surgical procedures, the tissue injury and systemic inflammatory response can trigger ROS production, exacerbating the overall stress response and potentially increasing postoperative complications [66].

Emerging evidence indicates that RA possesses antioxidant properties that can mitigate oxidative stress by inhibiting ROS production and preserving cellular homeostasis [52,67]. For instance, RA inhibits superoxide generation in neutrophils by reducing protein kinase C (PKC) activity, a key pathway involved in oxidative stress [68]. The attenuation of PKC and related pathways also helps reduce inflammatory cytokine release, further supporting RA’s role in limiting oxidative injury at the surgical site and systemically [69]. In a comprehensive analysis of regional anesthesia’s benefits, researchers have highlighted its potential to act as a protective agent against oxidative stress when combined with general anesthesia [70], potentially enhancing patient outcomes by reducing cellular damage and facilitating faster recovery antioxidant effects of RA, therefore complementing its other stress-attenuating mechanisms by reducing oxidative stress, contributing to a decrease in postoperative complications related to oxidative injury [71,72]. These findings support the role of RA in minimizing both inflammatory and oxidative components of the surgical stress response.

Beyond inflammatory markers, the stress response to surgery also involves a pronounced endocrine component, with cortisol and adrenaline being key mediators. Cortisol, a glucocorticoid hormone released in response to stress, exerts wide-ranging effects on immune modulation, metabolism, and tissue repair. Adrenaline, a catecholamine, is central to the “fight or flight” response and is associated with increased heart rate, blood pressure, and metabolic shifts. Elevated levels of these hormones are commonly observed following surgical procedures and are correlated with poor clinical outcomes, including impaired immune function, insulin resistance, and increased morbidity.

Several studies have shown that RA can significantly dampen this endocrine response. For instance, Xu et al. [73] conducted a randomized trial involving 165 patients. They demonstrated that administering a Transversus Abdominis Plane (TAP) block was associated with a marked reduction in cortisol and adrenaline levels. Patients receiving the TAP block exhibited significantly lower levels of these hormones in the perioperative period compared to those who did not receive RA, further illustrating the capacity of RA to modulate the systemic stress response. The ability of RA to suppress the release of cortisol and adrenaline is of particular clinical importance, as these hormones are directly linked to hyperglycemia, immunosuppression, and delayed wound healing—factors that contribute to increased postoperative complications and lengthened hospital stays.

The potential mechanisms underlying RA’s ability to reduce the secretion of stress-related hormones and inflammatory markers involve both neural and humoral pathways. By blocking afferent nociceptive input from the surgical site to the central nervous system (CNS), RA provides analgesia and prevents activating the hypothalamic–pituitary–adrenal (HPA) axis, thereby reducing cortisol secretion. Similarly, by modulating sympathetic nerve activity, RA decreases the release of catecholamines, such as adrenaline, from the adrenal medulla. This dual inhibition of inflammatory and endocrine pathways underscores the comprehensive impact of RA on the systemic stress response.

## 7. Comparative Outcomes: Regional Anesthesia (RA) vs. General Anesthesia (GA)

Comparative analyses from clinical studies demonstrate that RA offers statistically significant reductions in adverse outcomes across several domains:

- Incidence of Major Cardiovascular Events: RA has been associated with a reduced incidence of perioperative cardiac complications, including myocardial ischemia and arrhythmias, in contrast to GA, which may elevate these risks due to unmitigated stress-induced catecholamine surges and inflammatory responses [3,9,74,75].
- Mortality Rates: Data indicate a lower 30-day mortality rate in patients receiving RA, particularly among those undergoing high-risk procedures such as orthopedic and cardiovascular surgeries [76]. These findings are especially notable in elderly populations with more pronounced cardiovascular and immune vulnerabilities [77].
- Oncological Outcomes: Survival and Metastasis: In the context of oncological surgeries, RA has demonstrated the potential to positively influence cancer-related outcomes, such as long-term survival and metastasis rates [74,78]. RA’s immune-sparing and anti-inflammatory effects are pivotal in maintaining perioperative natural killer (NK) cell activity [79]. NK cells are crucial in targeting and eliminating circulating tumor cells, thereby reducing the risk of tumor recurrence and metastatic dissemination. By mitigating perioperative immunosuppression and inflammation, RA creates a less favorable environment for cancer cell proliferation, invasion, and metastasis [80,81].
- Reduction in Postoperative Complications: Patients receiving RA exhibit lower rates of postoperative complications, including pneumonia, atelectasis, and infections, and a reduced incidence of systemic inflammatory response syndrome (SIRS) [64]. This decrease may be attributed to RA’s ability to modulate inflammatory cytokine release, reduce immune suppression, and stabilize hemodynamics more effectively than GA [82].
- Length of ICU and Hospital Stay: RA has been consistently associated with a reduction in both ICU and total hospital length of stay [83]. This outcome is likely linked to RA’s efficacy in minimizing systemic inflammation, preserving immune function, and reducing the overall perioperative stress response [84]. Multiple studies report that RA results in ICU stays being shortened by an average of 1–2 days and hospital stays being reduced by 2–4 days when compared to GA [85].

Collectively, these outcomes indicate that RA addresses not only immediate perioperative analgesic needs but also contributes to a more stable postoperative trajectory [86]. With fewer complications, faster recovery times, and decreased healthcare resource utilization, RA presents a compelling case for inclusion as a standard protocol, particularly in high-risk and oncological surgeries [57,87]. The unique benefits observed in cancer patients receiving RA—reduced recurrence and metastasis rates—further reinforce its role in oncological anesthesia management, where the immune and anti-inflammatory preservation achieved by RA may prove crucial.

In oncological surgeries, RA’s role is critical in modulating cancer-related outcomes. Studies suggest that RA can reduce cancer metastasis risk and improve long-term survival by maintaining immune function [56]. Specifically, by reducing perioperative stress, RA preserves NK cell activity, which is essential for targeting residual cancer cells post-surgery [88]. RA’s reduction of inflammation and suppression of stress-induced immunosuppression also contribute to an environment less conducive to cancer cell proliferation and metastasis [89].

Comparative studies between RA and GA in cancer patients undergoing procedures such as breast, prostate, and colorectal surgeries show lower rates of cancer recurrence and metastasis in patients receiving RA [90,91]. RA’s anti-inflammatory and antioxidant effects and its neuroendocrine response inhibition reduce perioperative factors that otherwise promote tumor cell survival and spread [92].

## 8. Implications for Clinical Practice and Potential Shift in Anesthetic Standards

This review underscores the multi-system benefits of RA, suggesting it may surpass GA in enhancing surgical outcomes and reducing complications [93]. RA’s effectiveness in attenuating neuroendocrine, inflammatory, and oxidative stress responses makes it particularly valuable in managing the systemic stress response to surgery [94]. Integrating RA into perioperative care protocols, especially for high-risk patients and those undergoing oncological procedures, could offer surgical teams a means to enhance cardiovascular stability, support pulmonary function, maintain the immune response, and reduce recovery times [95,96].

This study contributes key insights by positioning RA as a cornerstone for comprehensive stress modulation beyond analgesia, highlighting its impact on inflammatory and oxidative pathways, which are essential for improved postoperative recovery and potentially long-term survival in cancer patients. By reducing reactive oxygen species (ROS) production and inflammation, RA positively influences wound healing, immune stability, and overall postoperative resilience. Furthermore, RA’s capacity to suppress stress-related hormones, such as cortisol and adrenaline, mitigates the risks associated with extended stress responses, including hyperglycemia, immunosuppression, and cardiovascular strain.

These findings suggest that RA could drive a shift in standard anesthesia practice, favoring its use as a fundamental component in multimodal anesthesia care to optimize both perioperative and oncological outcomes.

## 9. Conclusions

Regional anesthesia offers significant benefits in attenuating the systemic stress response to surgery. By modulating neuroendocrine, sympathetic, and inflammatory pathways, RA reduces the release of stress hormones, cytokines, and catecholamines, improving cardiovascular stability, reducing postoperative pain, and preserving immune function. These effects translate into improved clinical outcomes, particularly in high-risk patients such as the elderly or those with cardiovascular disease. Given the growing evidence supporting its efficacy, RA should be considered an essential component of multimodal perioperative care to reduce the systemic stress response and improve surgical and oncological outcomes. Further research, especially RCTs, is needed to optimize RA techniques and identify specific patient populations that may benefit most from this approach.

## Data Availability

The study datasets are available from the corresponding author at reasonable request.
